# Metabolism of *Lactobacillus sakei* Chr82 in the Presence of Different Amounts of Fermentable Sugars

**DOI:** 10.3390/foods9060720

**Published:** 2020-06-02

**Authors:** Federica Barbieri, Luca Laghi, Fausto Gardini, Chiara Montanari, Giulia Tabanelli

**Affiliations:** 1 Department of Agricultural and Food Sciences, University of Bologna, 47521 Cesena, Italy; federica.barbieri16@unibo.it (F.B.); l.laghi@unibo.it (L.L.); fausto.gardini@unibo.it (F.G.); 2 Interdepartmental Center for Industrial Agri-Food Research, University of Bologna, 47521 Cesena, Italy; giulia.tabanelli2@unibo.it; 3 Department of Agricultural and Food Sciences, University of Bologna, 40127 Bologna, Italy

**Keywords:** *Lactobacillus sakei*, sugar metabolism, amino acid metabolism, ^1^H-NMR, flow cytometry

## Abstract

*Lactobacillus sakei* is widely used as a starter culture in fermented sausages since it is well adapted to meat environments and able to maintain high viability thanks to secondary pathways activated when hexoses are depleted (i.e., metabolism of pentoses and amino acids). In this study, a commercial strain of *L. sakei* was inoculated in a defined medium with ribose or glucose as the carbon source, at optimal or reduced concentrations, to evaluate its different physiological and metabolic responses in relation to different growth conditions. The results obtained with different approaches (HPLC, ^1^H-NMR, flow cytometry) evidenced different growth performances, amino acid consumptions and physiological states of cells in relation to the carbon source as an active response to harsh conditions. As expected, higher concentrations of sugars induced higher growth performances and the accumulation of organic acids. The low sugars amount induced the presence of dead cells, while injured cells increased with ribose. Arginine was the main amino acid depleted, especially in the presence of higher ribose, and resulted in the production of ornithine. Moreover, the ^1^H-NMR analysis evidenced a higher consumption of serine at the optimal sugars concentration (pyruvate production). This information can be helpful to optimize the use of these species in the industrial production of fermented sausages.

## 1. Introduction

*Lactobacillus sakei* is a species with a high level of adaptation for meat environments in which it can rapidly grow and efficiently compete with other species present as components of the microbial communities of this raw material. Due to this aptitude, selected strains of this species are widely used as starter cultures in meat fermentation for dry sausages production [1]. The technological and safety advantage of the use of this species as a starter culture consists in its ability to inhibit pathogenic as well as spoilage microorganisms, to grow at a low temperature and to colonize the habitat during all of the ripening period, competing with undesired species [2]. This latter aspect, essential for guaranteeing the quality of fermented sausages throughout all the steps of production and commercialization, depends on its ability to efficiently produce metabolic energy even when the hexoses, which are fermented through the homofermentative pathway, are completely depleted. In fact, this species can also ferment pentoses contained in the nucleosides via the phosphoketolase pathway, as demonstrated by McLeod et al. [3] and Rimaux et al. [4].

Moreover, the arginine deiminase (ADI) pathway is active in *L. sakei*, even if with different efficiency. This pathway is an important additional energy source giving a competitive advantage in matrices with a low fermentable sugar concentration but high arginine content, such as meat [5,6].

The mean genotype size of *L. sakei* is relatively small (approximately 2020 kb) and reflects this specialization, even if a great variation in genome dimension is observed within the species (about 25%) [7]. The adaptation to grow in proteinaceous matrices (meat and fish) explains the absence of genes responsible for amino acid anabolism, in particular, transaminases: *L. sakei* strains are auxotrophic for all the amino acids, except for aspartate and glutamate [8]. Nevertheless, the metabolisms of some of these compounds are crucial for explaining the success of this species in the colonization of fermented meat.

In addition to the use of arginine for energy supply, other amino acids may be useful for the energetic strategies of this bacterium. Serine may be deaminated by L-serine dehydratase, yielding a surplus of pyruvate, and relevant uptakes of this molecule in defined media by *L. sakei* have been observed [5,6]. Threonine [5] and cysteine [6] were depleted in remarkable amounts (higher than those required by the generation of the intracellular amino acid pool) by this species under defined conditions. The presence of a gene coding for L-threonine dehydrogenase active in some *L. sakei* strains has been described: this protein catalyzes the conversion to glycine via 2-amino-3-ketobutyrate with a concomitant NAD^+^ reduction to NADH [5].

Survival and growth in environments poor in fermentable sugars have been also explained by an efficient pyruvate metabolism carried out for generating further ATP and gaining reducing power (regeneration of NAD^+^). The pyruvate formate lyase (PFL) pathway leads to the possible accumulation of by-products such as formate, acetate and ethanol in anaerobic or reducing conditions, while in an aerobic condition, CO_2_ and acetate may be produced through the pyruvate oxidase (POX) pathway and the pyruvate dehydrogenase complex (PDC). Enzymes involved in these pathways were found in *L. sakei* and their transcription was enhanced in the presence of pentoses as the fermentable substrate [9,10].

The same authors also demonstrated that glucose availability can affect different parameters such as growth rate, fermentative pathway (i.e., shift from homolactic to mixed acid fermentation), amino acid consumption and gene expression, but no effect on cell viability (in terms of percentage of alive cells) was observed. They hypothesized that this condition of low glucose availability is for *L. sakei* analogous to the so-called “complete caloric restriction”, that in eukaryotes, from single-celled yeast to humans, is a conserved mechanism that results in an expanded healthy life span in response to a reduction in energy intake [5].

A previous work was focused on the evaluation of the metabolic response of resting cells of six *L. sakei* strains in relation to the sugar presence (glucose or ribose) [6]. Cells in the stationary phase were inoculated at high concentrations (about 9 log CFU/mL) in a defined medium (DM) and incubated for 24 h to assess the consumption of sugars and amino acids and the resulting accumulation of organic acids and other metabolites. 

Based on the obtained results, the strain *L. sakei* Chr82 (used as a commercial starter culture in fermented sausage production) was chosen for the present work in order to study its growth, survival and metabolic response when inoculated at about 7 log CFU/mL in different DMs containing glucose or ribose at two initial concentrations (25 mM or 2.5 mM), to simulate an optimal or limited growth condition.

With the aim to better investigate the physiological response of this strain, different analytical approaches were used. In particular, cell cultivability was tested by plate count while flow cytometry was used to assess cell membrane permeability and depolarization as well as cell viability. Moreover, organic acid accumulation and amino acid variation were quantified by HPLC and the results were compared and discussed with metabolome analyses, performed by ^1^H-NMR. This latter aspect, that is the complete set of small metabolites consumed or produced [11], was done on the assumption that the metabolome would be the best representation of the microorganism phenotype, being downstream of the genome, transcriptome, and proteome. The analytical platform selected for this purpose was ^1^H-NMR, whose high reproducibility was expected to counterbalance the low sensitivity [12]. Moreover, this technique does not require derivatization or molecular separation and allows the untargeted, simultaneous detection of molecules pertaining to a broad range of chemical classes.

## 2. Materials and Methods

### 2.1. Microorganism Used

The commercial strain *Lactobacillus sakei* Chr82, supplied by the company Chr. Hansen (Parma, Italy), used as a starter culture in the production of fermented cured meats, was used.

### 2.2. Growth Media

*L. sakei* Chr82 was pre-grown in MRS broth prepared according to Oxoid formulation (peptone 10 g/L, lab-lemco powder 8 g/L, yeast extract 4 g/L, Tween 80 1 mL/L, dipotassium hydrogen phosphate 2 g/L, sodium acetate 3H_2_O 5 g/L, triammonium citrate 2 g/L, magnesium sulphate 7H_2_O 0.2 g/L, manganese sulphate 4H_2_O 0.05 g/L) and with the addition of two different sugars: in one case, 4.5 g/L of glucose, while in the other, 3.75 g/L of ribose were added. In the medium with ribose, according to the observation by McLeod et al. [3], a small amount of glucose was also added (0.2 g/L) in order to stimulate the growth of the microorganism in the initial phase.

The cells grown overnight under statically microaerophilic conditions at 30 °C in modified MRS were collected by centrifugation at 10,000 rpm for 10 min and suspended in a defined medium (DM), containing macro components, vitamins, nucleotides and amino acids. This DM, whose composition is reported in Table 1, is a modification of the medium proposed by Lauret et al. [13] for the growth of *L. sakei*. Amino acids were added at 0.2 g/L. The consequent mM concentration was as follows: alanine (ala) 2.24, arginine (arg) 1.15, asparagine (asg) 1.52, aspartic acid (asp) 1.50, cysteine (cys) 1.65, glutamic acid (glu), glutamine (glm) 1.36, glycine (gly) 2.66, histidine (his) 1.29, isoleucine (ile) 1.52, leucine (leu) 1.52, lysine (lys) 1.37, methionine (met) 1.34, phenylalanine (phe) 1.21, proline (pro) 1.74, serine (ser) 1.90, threonine (thr) 1.68, tryptophan (try) 0.98, tyrosine (tyr) 1.10, valine (val) 1.71.

The cells of *L. sakei* grown in the presence of glucose were suspended in DM with 2.5 mM of glucose and subsequently inoculated in the two DM added with glucose 2.5 mM (2.5 G) or 25 mM (25 G), at a cell concentration of about 7 log CFU/mL. The same procedure was applied for *L. sakei* Chr82 cells grown with ribose, but in this case the cells were suspended in DM with ribose 2.5 mM and then inoculated (cell load 7 log CFU/mL) in the two DM added with ribose 2.5 mM (2.5 R) or 25 mM (25 R). In the samples containing ribose, a small amount of glucose (0.1 mM) was added in order to provide the energy needed to activate the ribose metabolism related genes, as indicated by McLeod et al. [3]. The medium was sterilized by filtration at 0.22 µm (Sartorius Lab Instruments GmbH & Co. KG, Göttingen, Germany). The initial pH of the medium was 6.50 ± 0.02.

Inoculated samples were incubated at 30 °C and monitored at different times.

### 2.3. Growth Modeling and pH Measurement 

Growth performances were analyzed by measuring the increase in the optical density at 600 nm (OD_600_) using the UV–VIS spectrophotometer 6705 UV- Vis (Jenway, Stone, UK). Before each detection, a calibration of the instrument was performed with the blank (non-inoculated medium) of the respective sample.

The results of the optical density were modelled using the STATISTICA program (Statsoft Italia, Vigonza, Italy) through the Gompertz equation [14]:

$$y=k+A\cdot e^{-e^{\left[\left(\frac{{µ}_{max}\cdot e}{A}\right)\cdot \left(\lambda -t\right)+1\right]}}$$

where *y* is the OD_600_ at time *t*, *A* represents the maximum OD_600_ value reached, *μ_max_* is the maximum OD_600_ increase rate in the exponential phase and *λ* is the lag time.

The pH meter Basic 20 (Crison, Modena, Italy) was used for the sample pH measurement in order to monitor the acidification activity in the different conditions.

### 2.4. Microbiological Analysis

The microbiological counts of *L. sakei* Chr82 were carried out by plate counting in MRS agar (Oxoid, Basingstoke, United Kingdom) incubated aerobically for 48 h at 30 °C.

### 2.5. Organic Acids Content

The quantification of organic acids was performed using a HPLC instrument (PU-2089 Intelligent HPLC quaternary pump, UV-VIS multiwavelength detector UV 2070 Plus; Jasco Corp., Tokyo, Japan) equipped with a manual Rheodyne injector with a 20 μL loop (Rheodyne, Rohnert Park, CA, USA) and a Bio-Rad Aminex HPX-87H column with a size of 300 × 7.8 mm (Bio-Rad Laboratories, Hertfordshire, UK).

The analysis was performed in isocratic conditions at 65 °C with a rate flow of 0.6 mL/min of mobile phase H_2_SO_4_ 0.005 M. The UV detector was set at 210 nm. Chromatographic peaks were identified by comparing retention times with those of standards (Sigma-Aldrich, St. Louis, MO, USA) and quantification was carried out by using the external standard method.

### 2.6. Quantification of Amino Acids

To evaluate the variation in the amino acid concentration, samples were analyzed by HPLC (PU-1580 Intelligent HPLC, Intelligent Fluorescence Detector FP-1520 and Intelligent Sampler AS-2055 Plus, with 10 μL loop; Jasco Corp., Tokyo, Japan), after a derivatization using an AccQ-Fluor Reagent kit (Waters Corp., Milford, MA, USA) according to the method described by Montanari et al. [6].

The separation of amino acids was performed using an AccQ-Tag_TM_ column (3.9 × 150 mm; Waters Corp.) at 30 °C using mobile phase A (100 mL of AccQ-Tag Eluent (Waters Corp., Milford, MA, USA), diluted 1:10 with H_2_O for chromatography (Sigma-Aldrich, St. Louis, MO, USA) and mobile phase B (60% acetonitrile and 40% H_2_O for chromatography (Sigma-Aldrich, St. Louis, MO, USA)) at a flow rate of 1 mL/min. The fluorescent detector was set at an excitation wavelength of 250 nm and emission wavelength of 395 nm. Under the adopted conditions, good separation of the amino acids was obtained with the exception of the couples histidine + glutamine and serine + asparagine, which coeluted in unique peaks. Tryptophan was not detectable with this protocol.

### 2.7. Flow Cytometric Analysis

Flow cytometry (FCM) was used to monitor the physiological state of *L. sakei* Chr82 cells in each sample. Cell suspensions were analyzed with the flow cytometer Accuri C6 (BD Biosciences, Milan, Italy), using setting parameters, emission filters and thresholds according to Arioli et al. [15].

Before the analysis, where necessary, the samples were diluted in the corresponding DM up to a concentration of 7 log CFU/mL, the optimal cell density for a correct sample staining by fluorochromes.

The cells were stained with SYBR-Green I (1X), propidium iodide (PI) 7.5 μM and DiBAC_4_ (3) (Bis-(1,3-Dibutylbarbituric Acid) Trimethine Oxonol) 3.0 μM as reported by Tabanelli et al. [16]. The data obtained were analyzed using the BD ACCURITM C6 software version 1.0 (BD Biosciences, Milan, Italy). Before analysis, each aliquot was kept at 37 °C for 15 min in order to let the dye react with the cells.

### 2.8. Untargeted Metabolomics Analysis by ^1^H-NMR

For the metabolomics investigation by ^1^H-NMR, an analysis solution was created, with 3-(trimethylsilyl)-propionic-2,2,3,3-d4 acid sodium salt (TSP) 10 mM in D_2_O, set at pH 7.00 ± 0.02 by means of 1 M phosphate buffer. The solution contained also 10 μL of NaN_3_ 2 mM, to avoid microbial proliferation, while TSP was employed as the ^1^H NMR chemical-shift reference, as suggested by Zhu et al. [17]. Growth medium samples were prepared for ^1^H-NMR by thawing and centrifuging 1 mL of each for 15 min at 18,630 g and 4 °C. An amount of 700 μL of supernatant was added to 200 μL of the ^1^H NMR analysis solution. Finally, each of the so-obtained samples was centrifuged again at the above conditions right before analysis.

^1^H-NMR spectra were recorded at 298 K with an AVANCE III spectrometer (Bruker, Milan, Italy), operating at a frequency of 600.13 MHz, equipped with the software Topspin 3.5. Following the procedure described by Laghi et al. [12], the HOD residual signal was suppressed by applying the first increment of the nuclear overhauser effect spectroscopy (NOESY) pulse sequence and a spoil gradient. This was done by employing the NOESYGPPR1D sequence, part of the standard pulse sequence library. Each spectrum was acquired by summing up 256 transients using 32 K data points over a 7184 Hz spectral window, with an acquisition time of 2.28 s. The spectra were phase- and baseline-adjusted in Tospin, that was employed also for the calculation of the signal-to-noise ratio. Spectra were elaborated with the R package (R Core Team, 2018, Vienna, Austria) as reported by Zhu et al. [17]. Molecules identification was performed by comparing their signals with those of pure compounds by the Chenomx software ver. 8.3 (Chenomx Inc., Edmonton, AB, Canada) with the Chenomx (ver. 10) and HMDB (release 2) libraries.

## 3. Results and Discussion

### 3.1. Determination of Growth Curves and Microbiological Analysis

The DM containing the two different sugars (glucose and ribose) at the two different concentrations (2.5 and 25 mM) were inoculated with approximately 7 log CFU/mL of *L. sakei* Chr82. The experimental data of the growth dynamics monitored by measuring the OD_600_ were modelled with the Gompertz equation [14] and are reported in Supplementary Materials Figure S1. The growth parameters obtained, together with the cell concentrations after 24 and 48 h of incubation, are summarized in Table 2. The amount of sugars influenced the maximum OD_600_, which reached values of 0.264 and 0.282 in the presence of glucose and ribose at 2.5 mM, respectively, while, under the same conditions, the maximum OD_600_ predicted when the sugars were added at 25 mM were 1.446 and 1.151. The addition of ribose determined a slightly longer *λ* time and lower µ_max_. Regarding the cell concentrations after 24 and 48 h of growth, determined by plate counting, no significant differences were found after 24 h in the different media (Table 2). After 48 h, cell loads showed a drastic decline (1 log unit or more) if compared with the counts at 24 h and the higher survival rate was observed in the sample containing 2.5 mM·R. The rapid beginning of the death phase in culture media for this species has already been observed [18] and it is in contrast with the long survival showed by *L. sakei* in stricter conditions as those characterizing fermented sausages during ripening. 

### 3.2. Organic Acid Content and pH

In Table 3, the organic acids accumulated after 24 and 48 h of incubation and detected by HPLC are reported.

Considering the lower sugar concentration (2.5 mM), only L-lactate was detected in these samples. In the presence of glucose, its presence (approximately 4 mM) was accompanied by a lower proportion of acetate (0.52 and 0.62 mM after 24 and 48 h, respectively). Higher amounts of acetate (approximately 3 mM) were produced, as expected, in the presence of ribose. In addition, the molar production of the two acids represented more that 90% of the theoretical yield. In the presence of ribose, the ratio acetate/lactate was higher than 1, indicating the activation of pathways alternative to homolactic and heterolactic fermentations [19].

The presence of fermentable carbohydrates at 25 mM determined the production of more than 40 mM of lactate and small amounts (2 mM) of acetate in the medium added with glucose, while the addition of ribose resulted in the accumulation of lactate (more than 17 mM, including L- and D-lactate) and relevant quantities of acetate (more than 21 mM). In this latter case, the quantitative production of acetate was higher than expected (acetate/lactate molar ratio higher than 1) as a consequence of the activity of secondary pathways. As observed by McLeod et al. [9], *L. sakei* alters its pyruvate metabolism when grown in the presence of ribose, generating more ATP per ribose unit up-regulating pyruvate decarboxylases and pyruvate dehydrogenases which can bring to the accumulation of acetate [19]. 

The presence of glucose compared with ribose always determined a lower pH (Table 3), and after 48 h, values of 5.83 vs. 6.21 were found with the addition of the sugars at 2.5 mM, while at 25 mM, the pH values measured were 4.00 and 4.37 for glucose and ribose, respectively.

### 3.3. Amino Acids Quantification

It is well known that *L. sakei*, because of its adaptation to meat environments, is auxotrophic for 18 amino acids [8]. The study of the variations in the amino acid content in a defined medium is important to elucidate how this species uses these molecules. In the first instance, they are used to assemble all the proteins (and enzymes) necessary to sustain growth and multiplication. However, it is interesting to evidence amino acid alternative uses in the perspective of explaining the high persistence of *L. sakei* cells in habitats, such as fermented sausages, in which fermentable sugars are rapidly depleted. In a previous work, Montanari et al. [6] described amino acid variations due to the resuspension of resting cells in a defined medium. In the present research, the changes in amino acid concentration are studied after *L. sakei* growth, and the influence of cell metabolism on each amino acid, as revealed by HPLC analyses, is shown in Table 4.

**Figure 1:**
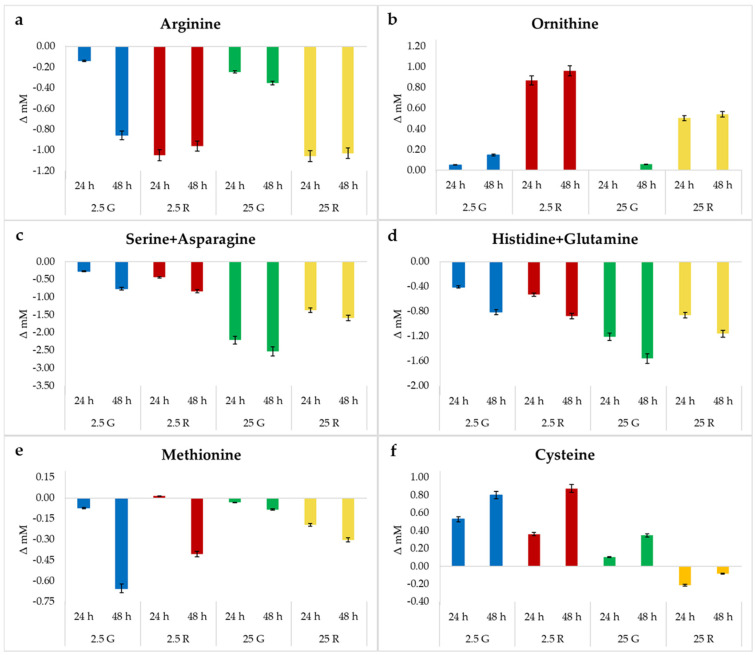
Variation in the amounts of amino acids characterized by the most relevant modification after 24 and 48 h of incubation at 30 °C of *L. sakei* Chr82 with respect to the initial concentration in the DM, and ornithine production. The standard deviations are reported.

The concentration of many amino acids after 24 and 48 h of incubation showed small variations with respect to the initial level. Aspartate, alanine, valine, lysine and leucine were always consumed in an amount lower than 20% of the initial concentration. Smaller variations were observed for isoleucine, tyrosine, threonine and glycine. Phenylalanine was accumulated (up to 17% of the initial concentration) in all the conditions tested with a trend similar to that observed by McLeod et al. [5]. Glutamic acid, generally consumed in all the other conditions, was accumulated in the presence of glucose 25 mM. The remaining amino acids were subjected to more relevant variations (Figure 1).

The use of arginine to produce ATP through the ADI pathway has been well studied in *L. sakei* [20]. However, different patterns of decrease have been observed among strains [5,6]. In this case, glucose retarded arginine depletion, even if after 48 h, the presence at 2.5 mM of this sugar caused a high consumption of this amino acid (Figure 1a). By contrast, the presence of ribose as a fermentable sugar determined a rapid and massive consumption of this amino acid, independently of the ribose concentration.

The activation of the ADI pathway is considered crucial to allow the survival of this species in meat environments. Some strains possess a second putative ADI pathway, which improves their ability to take advantages from the high amounts of arginine in meats [5]. This activity is confirmed by the accumulation of ornithine (Figure 1b). Ornithine is the final product of the ADI pathway and was produced in higher amounts in the presence of ribose, particularly 2.5 mM. The absence of a correspondence between arginine consumption and ornithine production could be attributed to the ability of this strain to decarboxylate this amino acid. In fact, only *L. sakei* Chr82, among the six strains tested by Montanari et al. [6], was able to produce putrescine from the decarboxylation of ornithine. However, in the conditions adopted in the present study, this biogenic amine was never detected.

The sum of serine and asparagine (not separated under the adopted HPLC analytical protocol) showed a drastic decrease, especially in the samples containing a high sugar concentration and after 48 h of incubation (Figure 1c). Serine can be used to supply pyruvate, which can then be used to produce energy through the PFL or POX pathways [21]. The conversion of serine into pyruvate has been described in *Pediococcus pentosaceus* as the result of the activity of a serine dehydratase [22], while *Lactobacillus plantarum* could metabolize serine with the production of formate, succinate and acetate [23]. McLeod et al. [5] showed a high use of serine and asparagine in *L. sakei* strains grown under glucose limiting conditions. Among the six *L. sakei* strains tested by Montanari et al. [6] under resting conditions, the strain Chr82 was the most efficient in serine + aspargine uptake in the absence of fermentable sugars. The decrease was higher in the sample containing glucose, in contrast to the trend observed for arginine.

Further, the consumption of histidine + glutamine (not separated under the adopted HPLC analytical protocol) was higher in the media containing glucose (Figure 1d). The resuspension of resting cells of the same strain in the DM did not markedly change the concentration of these amino acids. By contrast, growing cells decreased the concentration of these amino acids, especially after 48 h and when glucose was present in the medium. McLeod et al. [5] showed a strong decrease in glutamine during a continuous cultivation in a glucose-limited medium inoculated with two *L. sakei* strains, while the concentration of histidine was scarcely affected.

Finally, the sulfur amino acids methionine and cysteine showed a correlated trend (Figure 1e,f). Relevant diminutions of methionine were observed after 48 h in the samples with 2.5 mM of both sugars. However, these decreases were accompanied by concomitant increases in cysteine. Only the samples containing ribose 25 mM presented a simultaneous decrease in both amino acids.

### 3.4. Flow Cytometric Analysis

**Figure 2:**
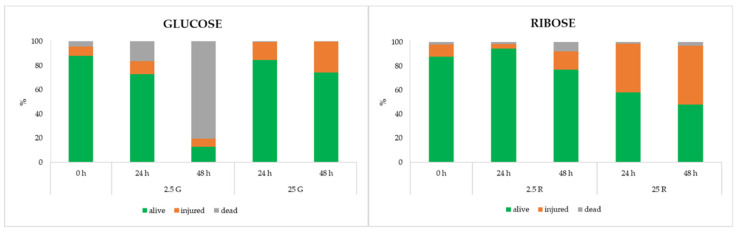
Distribution of alive, damaged and dead cells of *L. sakei* Chr82 after 24 and 48 h of incubation at 30 °C in different DMs. The data are reported as the relative frequency of the total population obtained by flow cytometric (FCM) analysis with dual staining (SYBR-Green I and PI).

The same samples were also subjected to flow cytometric (FCM) analysis to define some parameters linked to cell viability. Each sample was labeled with SYBR-Green I and propidium iodide (PI) in a 1:1 ratio. This dual staining allowed to discriminate three sub-populations: alive, damaged or dead cells. The results are shown in Figure 2.

In the samples containing glucose, a higher viability in the cells grown in the presence of 25 mM of this sugar was generally observed. The percentage of cells recognized as alive was 84.6% and 74.4%, after 24 and 48 h, respectively. At the same sampling times, the injured cells passed from 7.6% to 14.9% and 25.5% and dead cells from 4.28% to 0.5% and 0.1%, respectively. The presence of a limited amount of glucose (2.5 mM) determined a drastic increase in the dead cells (approximately 18% after 24 h and 80% after 48 h). When the ribose was added at the higher concentration (25 mM), the number of dead cells remained comparable to those observed in the presence of glucose, but the portion of damaged cells was much more relevant, already starting from 24 h of incubation. Conversely, the addition of ribose 2.5 mM resulted, after 48 h, in a higher viability if compared with the sample added with the same amount of glucose.

**Figure 3:**
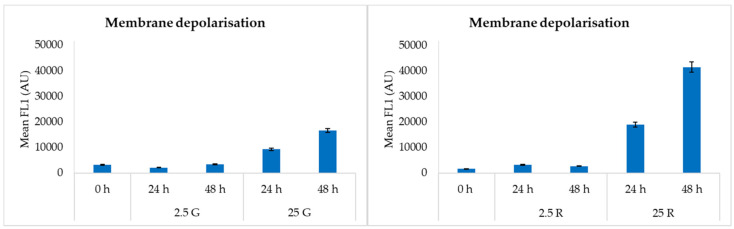
FCM analysis related to membrane depolarization in *L. sakei* Chr82 after 24 and 48 h of incubation at 30 °C in different DMs. Data are reported as mean fluorescence of DiBAC_4_ (3) dye (arbitrary unit, AU).

Regarding the membrane depolarization, expressed as fluorescence of DiBAC_4_ (3), the results obtained for the sugars at the two concentrations (Figure 3) showed that this parameter is inversely proportional to the media pH. In fact, in the presence of the higher sugar concentrations, and therefore with the lowest media pH values (4 in the presence of glucose and 4.6 in the presence of ribose), cell membrane depolarization was greater. In the 2.5 G and 2.5 R samples, characterized by higher pH values (about 5.8 in the presence of glucose and 6.2 in the presence of ribose), the depolarization degree was lower.

**Figure 4:**
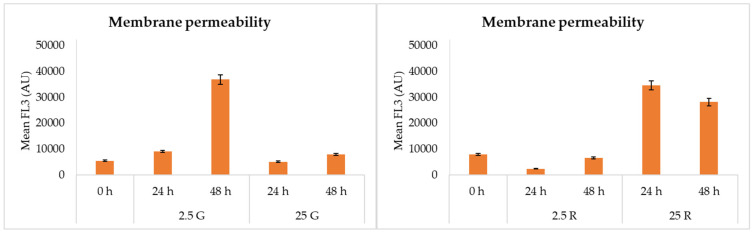
FCM analysis related to membrane permeability in *L. sakei* Chr82 after 24 and 48 h of incubation at 30 °C in different DMs. Data are reported as mean fluorescence of the propidium iodide (PI) (arbitrary unit, AU).

Finally, the results of membrane permeability (Figure 4) showed higher values in the presence of glucose 2.5 mM, while the trend was opposite in the presence of ribose, where the membrane permeability was higher in the presence of the higher concentration of this sugar.

### 3.5. Untargeted Metabolomics Analysis by ^1^H-NMR

**Figure 5:**
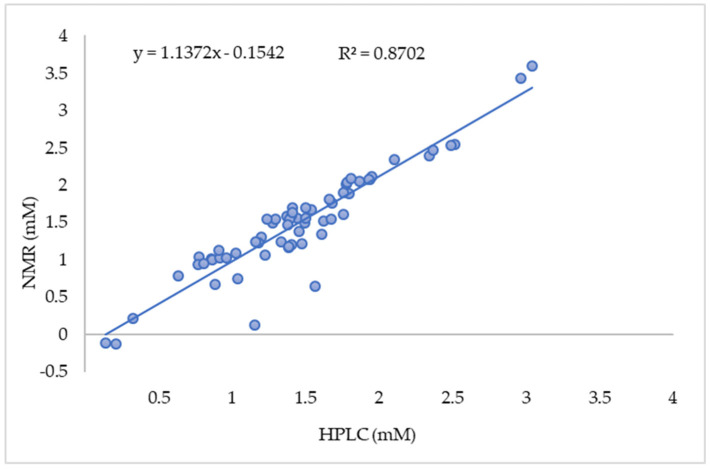
Correlation matrix between ^1^H-NMR and HPLC results (expressed as mM). In the figure are reported the correlation line between NMR results (y) and HPLC results (x) and the correlation coefficient (R^2^).

With the aim to have a deeper insight in the metabolomic responses of the strain *L. sakei* Chr82, the same samples were further analyzed applying a quantification protocol based on ^1^H-NMR. An example of portions of the ^1^H-NMR spectrum obtained from one representative sample is reported in Supplementary Materials Figure S2. Regarding the amino acid concentration, the correlation between the NMR and HPLC results was satisfying, as demonstrated by the regression analysis reported in Figure 5, characterized by a high R^2^ (0.8702), an intercept of 0.1542 and an angular coefficient close to 1 (1.1372). Previous works supported the suitability of the ^1^H-NMR approach: for example, Biagioli et al. [24] were able to observe divergent metabolic activities of two batches of the same probiotic preparation; Parolin et al. [25] identified the metabolome traits distinguishing vaginal lactobacilli with different anti-candida activity; and Picone et al. [26] followed the adaptation of *Escherichia coli* 555 to increasing doses of carvacrol.

**Figure 6:**
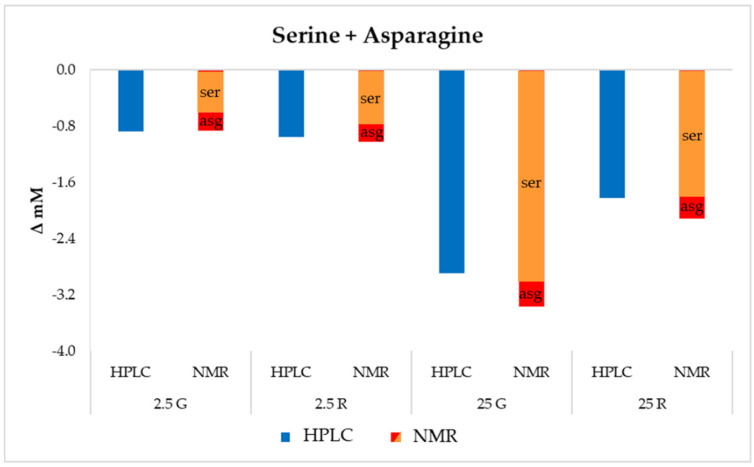
Variation (mM) in serine and asparagine with respect to the initial concentration in the different DMs after 48 h of incubation.

**Figure 7:**
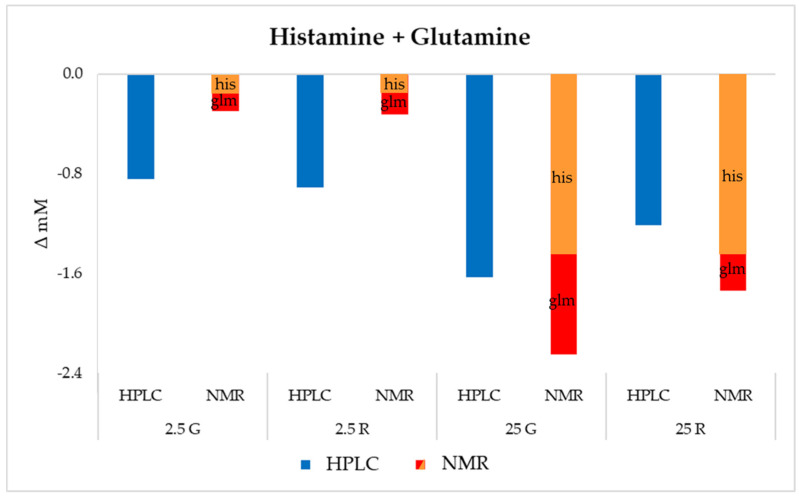
Variation (mM) in histidine and glutamine with respect to the initial concentration in the different DMs after 48 h of incubation.

NMR data were focused, at a first instance, to the evaluation of the amino acids not separated by the HPLC analysis, i.e., serine + asparagine and histidine + glutamine. The results regarding these amino acids, expressed as concentration variation, are reported in Figure 6 and Figure 7.

Taking into consideration serine + asparagine, the total decrease in these two molecules detected with the two methods were comparable. However, the ^1^H-NMR approach indicated that the main decrease concerned serine (Figure 6), confirming the hypothesis that this amino acid can provide a supply of pyruvate, which can be addressed to alternative metabolic pathways that are important for when available sugars become a limiting factor. Liu et al. [27] proved the central role of pyruvate deriving from serine in *L. plantarum* metabolism, demonstrating that it was involved in the regeneration of NADH and in the production of ATP, acetate, formate, ethanol, acetoin, diacetyl and 2-3-butanediol. 

Lower concordance was observed for the data of histidine + glutamine (Figure 7). While in the presence of a low sugar concentration, the ^1^H-NMR analysis reported lower amounts of amino acids uptake if compared with the HPLC results, and an opposite trend characterized the samples with the higher glucose and ribose concentration. In any case, according to the ^1^H-NMR results, the consumption of the two amino acids was reduced at a similar rate in the presence of 2.5 mM of the sugars, while, when the sugars were present at high concentration, histidine was depleted much more than glutamine.

In addition to the amino acid content, the untargeted ^1^H-NMR protocol applied allowed the detection of other metabolic compounds (Table 5).

Regarding sugars, they resulted in being completely depleted in the media in which they were added at 2.5 mM, while in the samples added with 25 mM, small residual quantities (approximately 0.15 mM) were detected. Further, ethanol was detected in extremely low amounts, indicating that the secondary metabolic pathways activated by *L. sakei* Chr82 were mainly addressed towards the production of acetic acid which allows the production of energy rather than the regeneration of reduced NADH. The production of acetoin and 2,3-butanediol was higher in the samples containing a higher sugar concentration, particularly in the sample with ribose.

## 4. Conclusions

The results obtained in this work increase the knowledge on the physiological and metabolic responses of *L. sakei* in relation to different sugar amounts. Indeed, the combined use of HPLC and ^1^H-NMR approaches allowed to better elucidate the consumption of amino acids and the resulting metabolites produced during incubation.

As expected, higher concentrations of glucose or ribose induced higher growth performances, acidification of the growth medium and accumulation of lactic and acetic acids.

The flow cytometric analysis evidenced a different physiological adaptation to the conditions; in fact, even if cells grown on glucose at a high concentration had a high viability, the same sugar in low amounts induced the presence of dead cells, while ribose determined the higher percentage of injured cells, but only few cells were recognized as dead also when this pentose was present at 2.5 mM.

The analysis of amino acids confirmed the rapid depletion of specific amino acids, mainly arginine, whose consumption was higher in the presence of ribose and resulted in the production of ornithine. Other amino acids highly consumed by this strain were serine, asparagine, glutamine and histidine. Since the HPLC protocol adopted was not able to separate these metabolites (co-elution of serine + asparagine and glutamine + histidine), ^1^H-NMR analysis in this case was helpful to discriminate the single metabolites, allowing to evidence a higher consumption of serine, especially when sugars were present at an optimal concentration. This consumption of serine confirmed other findings reported in the literature for lactic acid bacteria, i.e., the use of this amino acid as a source of pyruvate, which can then be used to produce energy through secondary pathways.

The untargeted ^1^H-NMR analysis performed on the samples with the aim to set up a fast method to simultaneously quantify amino acids, sugars, organic acids and other molecules was successful. This approach resulted as indeed suitable and very promising to evaluate the metabolic response of *L. sakei* in terms of the consumption and accumulation of specific metabolites.

This information can be helpful to optimize the use of this species as a starter culture for the industrial production of fermented sausages, since stressful conditions can affect the microbial technological performances or induce the activation of specific metabolic pathways, whose final products can have a significant impact on the sensorial features of the fermented sausages obtained.

## Figures and Tables

**Table 1:** Chemically defined medium (DM) composition.

Compound	Concentration (g/L)
**Macro components**	
Sodium acetate	2.0
K_2_HPO_4_	1.75
MnSO_4_ 4H_2_O	0.012
MgSO_4_ 7H_2_O	0.2
Tween 80	1 mL
**Vitamins**	
Thiamine HCl	0.0011
Folic acid	0.0002
Riboflavin	0.001
Calcium pantothenate	0.001
Nicotinic acid	0.001
Pyridoxal-5-phosphate	0.0005
p-amino benzoic acid	0.0004
**Nucleotides**	
Adenine	0.005
Guanine	0.01
Uracil	0.01

**Table 2:** Cell load (expressed as log CFU/mL) after 24 and 48 h of incubation of *L. sakei* Chr82 at 30 °C in the different DM and growth parameters obtained modelling the growth dynamics in different DM (measure of OD_600_) with the Gompertz equation.

Sample	Time	Cell Counts	Growth Parameters (Gompertz Equation)			
		(Log CFU/mL)	*k*	*A*	*µ_max_*	*λ*
	0 h	6.94 (±0.21)				
**2.5 G**	24 h	8.15 (±0.23)	0.044	0.264	0.155	11.457
	48 h	6.67 (±0.11)				
**2.5 R**	24 h	8.41 (±0.16)	0.046	0.282	0.120	11.570
	48 h	7.62 (±0.13)				
**25 G**	24 h	8.13 (±0.21)	0.045	1.446	0.280	10.839
	48 h	6.98 (±0.13)				
**25 R**	24 h	8.10 (±0.11)	0.074	1.151	0.275	11.257
	48 h	6.89 (±0.14)				

**Table 3:** Organic acid content and pH values of *L. sakei* Chr82 samples incubated at 30 °C in the different DMs. Acetic acid concentration is expressed as a difference with respect to the initial amount added in the media as sodium acetate (24 mM).

Sample	Time	L-Lactic Acid (mM)	D-Lactic Acid (mM)	Acetic Acid (mM)	pH
2.5 G	24 h	4.25 (±0.09)	- *	0.52 (±0.20)	5.78 (±0.29)
	48 h	3.88 (±0.14)	-	0.62 (±0.15)	5.83 (±0.16)
2.5 R	24 h	1.66 (±0.07)	-	2.85 (±0.11)	6.17 (±0.30)
	48 h	1.39 (±0.04)	-	3.15 (±0.09)	6.21 (±0.27)
25 G	24 h	39.60 (±1.98)	0.41 (±0.09)	2.58 (±0.03)	4.15 (±0.17)
	48 h	43.81 (±2.05)	0.39 (±0.10)	2.77 (±0.28)	4.00 (±0.22)
25 R	24 h	15.61 (±0.90)	1.43 (±0.20)	21.00 (±0.43)	4.73 (±0.21)
	48 h	16.02 (±0.63)	1.09 (±0.14)	22.70 (±0.67)	4.37 (±0.24)

* Under the detection limit (0.1 mM).

**Table 4:** Amino acid concentrations (mM) detected by HPLC in DM after 24 and 48 h of incubation of *L. sakei* Chr82 at 30 °C in the different DMs. In brackets, the relative variations (as percentage) with respect to the initial concentration are reported.

		Asp	Ser + Asg	Glu	Gly	His + Glm	Arg	Thr	Ala	Pro	Cys	Tyr	Val	Met	Lys	Ile	Leu	Phe
Initial concentration		1.50	3.42	1.36	2.66	2.66	1.15	1.68	2.24	1.74	1.65	1.10	1.71	1.34	1.37	1.52	1.52	1.21
Sample	Time																	
2.5 G	24 h	1.39 (−7.49)	3.15 (−7.94)	1.27 (−6.53)	2.91 (9.53)	2.25 (−15.27)	1.01 (−12.00)	1.79 (6.57)	2.12 (−1.09)	1.92 (10.49)	2.18 (32.02)	1.11 (0.85)	1.84 (7.68)	1.27 (−5.45)	1.36 (−0.45)	1.50 (−1.54)	1.39 (−8.51)	1.28 (6.02)
	48 h	1.27 (−15.53)	2.65 (−22.41)	1.07 (−21.36)	2.50 (−5.97)	1.85 (−30.44)	0.29 (−74.57)	1.80 (7.29)	2.03 (−9.22)	1.80 (3.20)	2.45 (48.56)	1.04 (−5.83)	1.66 (−2.90)	0.69 (−48.88)	1.44 (4.96)	1.53 (0.68)	1.49 (−2.22)	1.41 (16.51)
2.5 R	24 h	1.32 (−12.26)	2.98 (−13.00)	1.18 (−13.50)	2.81 (5.61)	2.13 (−19.94)	0.10 (−91.57)	1.65 (−1.87)	2.21 (−1.38)	1.78 (2.09)	2.01 (21.78)	1.10 (0.12)	1.69 (−0.96)	1.36 (1.18)	1.36 (−0.67)	1.58 (3.63)	1.43 (−6.03)	1.26 (4.53)
	48 h	1.44 (−11.04)	2.58 (−24.42)	1.08 (−20.47)	2.48 (−6.82)	1.79 (−32.85)	0.19 (−83.76)	1.71 (2.08)	2.02 (−10.03)	1.77 (1.75)	2.53 (53.09)	1.14 (3.93)	1.62 (−5.05)	0.94 (−30.22)	1.41 (2.99)	1.53 (0.39)	1.50 (−1.58)	1.34 (10.72)
25 G	24 h	1.30 (−4.00)	1.21 (−64.72)	1.73 (27.56)	2.71 (1.78)	1.45 (−45.33)	0.90 (−21.38)	1.56 (−6.93)	1.92 (−14.09)	1.69 (−3.07)	1.75 (6.31)	1.07 (−2.87)	1.59 (−7.27)	1.31 (−2.42)	1.23 (−9.96)	1.59 (4.93)	1.39 (−8.25)	1.42 (17.71)
	48 h	1.41 (−13.27)	0.89 (−73.95)	1.63 (19.88)	2.33 (−12.43)	1.10 (−58.54)	0.80 (−30.78)	1.36 (−19.13)	1.73 (−22.80)	1.75 (0.81)	2.00 (21.05)	1.02 (−7.05)	1.50 (−12.17)	1.26 (−6.27)	1.10 (−19.61)	1.39 (−8.81)	1.45 (−4.67)	1.32 (9.38)
25 R	24 h	1.28 (−14.55)	2.05 (−40.17)	1.33 (−2.17)	2.62 (−1.41)	1.80 (−32.31)	0.09 (−92.08)	1.77 (5.53)	2.02 (−9.78)	1.75 (0.30)	1.43 (−13.05)	1.01 (−7.88)	1.59 (−6.93)	1.15 (−14.50)	1.21 (−11.82)	1.49 (−2.04)	1.46 (−4.22)	1.35 (11.82)
	48 h	1.20 (−20.06)	1.83 (−46.41)	1.18 (−13.32)	2.35 (−11.60)	1.50 (−43.72)	0.12 (−89.31)	1.64 (−2.14)	1.86 (−17.08)	1.80 (3.60)	1.57 (−5.09)	1.08 (−2.27)	1.52 (−11.15)	1.04 (−22.76)	1.09 (−20.45)	1.34 (−11.72)	1.37 (−9.93)	1.32 (9.45)

**Table 5:** Concentration (expressed as mM) of some metabolic compounds detected by ^1^H-NMR after 48 h of *L. sakei* Chr82 incubation at 30 °C in different DMs.

	Ethanol	Acetoin	2,3-Butanediol	Glucose	Ribose
2.5 G	0.01	0.10	0.01	- *	-
2.5 R	0.01	0.15	0.01	-	-
25 G	0.03	0.14	0.06	0.14	-
25 R	0.01	0.21	0.19	-	0.15

* Under the detection limit (0.01 mM).

## Supplementary Materials

The following are available online at https://www.mdpi.com/2304-8158/9/6/720/s1, Figure S1: Growth curves of *L. sakei* Chr82 at 30 °C under different conditions. In the square the Gompertz parameters are reported. Points: experimental data; lines: fitting curves as predicted by Gompertz equation, Figure S2. Portions of 1H-NMR spectrum from *L. sakei* Chr82 inoculated in DM with 25 mM of glucose reporting, for the molecules listed in Table 4 and Table 5, the signals employed for quantification. The exact extremes of the signals are represented by dashed lines (p. 14).
