# Supplementary material for: Metabolism of Lactobacillus sakei Chr82 in the Presence of Different Amounts of Fermentable Sugars
Source: Foods. 2020 Jun 2;9(6):720. doi: 10.3390/foods9060720 (PMC7353496; doi:10.3390/foods9060720)
Supplement: Supplementary file 1 [file foods-09-00720-s001.docx]

**Supplementary material**

**Figure S1.** Growth curves of *L. sakei* Chr82 at 30°C under different conditions. In the square the Gompertz parameters are reported. Points: experimental data; lines: fitting curves as predicted by Gompertz equation.


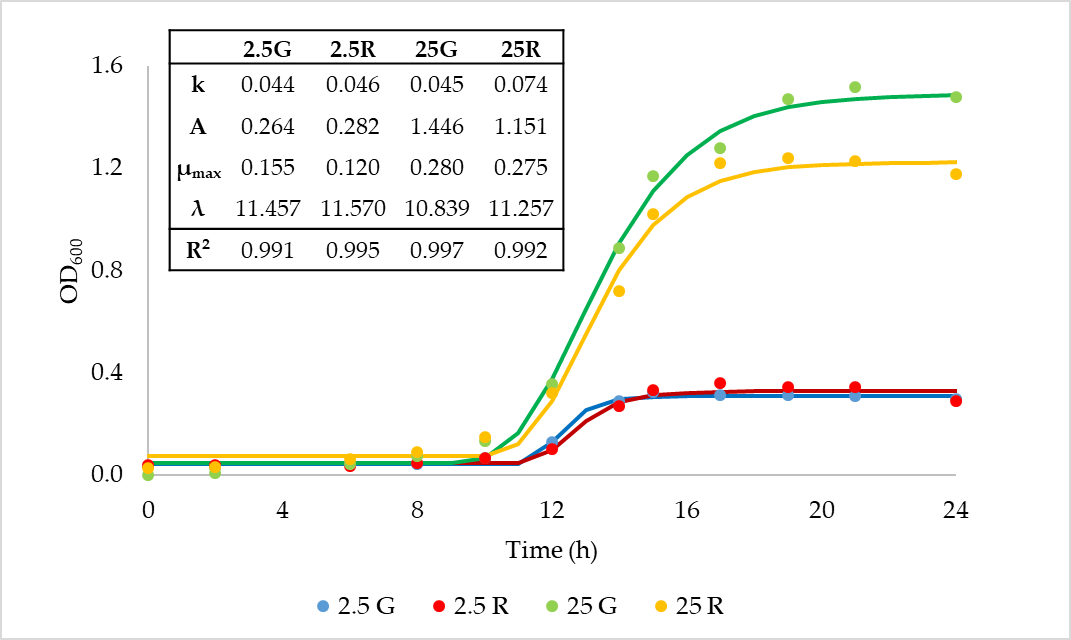


**Figure S2.** Portions of 1H-NMR spectrum from *L. sakei* Chr82 inoculated in DM with 25 mM of glucose reporting, for the molecules listed in Tables 4 and 5, the signals employed for quantification. The exact extremes of the signals are represented by dashed lines.
